# Atrophic Dermatofibroma: A Rare Variant With Distinct Clinical and Histopathological Features

**DOI:** 10.1155/crdm/4419249

**Published:** 2026-10-21

**Authors:** Renad F. Althobaiti, Jana Khalid Alharbi, Asmaa Alabbadi, Dai Zafer, Razan Alluhaibi, Bayan Zakaria Hafiz

**Affiliations:** ^1^ College of Medicine Umm Al-Qura University Makkah Saudi Arabia uqu.edu.sa; ^2^ Dermatology Department King Abdulaziz Hospital Makkah Saudi Arabia mngha.med.sa; ^3^ Dermatology Department Makkah Health Cluster Makkah Saudi Arabia; ^4^ Department of Pathology Makkah Health Cluster Makkah Saudi Arabia

**Keywords:** atrophic dermatofibroma, cutaneous soft-tissue tumor, dermatofibroma, fibrous histiocytoma

## Abstract

Dermatofibroma (DF) is a common benign cutaneous soft‐tissue tumor that typically affects females between the third and fifth decades of life. While its pathogenesis is not fully understood, DF has been associated with minor local trauma and insect bites. We present a case of atrophic DF in a 35‐year‐old Saudi female who noticed a solitary, hyperpigmented, depressed plaque on her back that had been present for five years. The lesion initially appeared as a small papule but gradually increased in size while remaining asymptomatic, without pain, itching, or systemic complaints. Physical examination revealed a well‐demarcated, hyperpigmented, depressed plaque without ulceration or surrounding erythema. Histopathological examination supported the diagnosis of atrophic DF, and immunohistochemical staining showed focal positivity for Factor XIIIa and negative staining for CD34, S100, HMB45, and p63. Atrophic DF is a rare variant characterized by a depressed clinical appearance and distinctive histopathological features that may create diagnostic challenges. Recognition of this uncommon presentation and correlation of the clinical, histopathological, and immunohistochemical findings can help distinguish it from other atrophic and spindle‐cell cutaneous lesions.

## 1. Introduction

Dermatofibroma (DF) is a common benign cutaneous neoplasm with a broad spectrum of clinical and histopathological appearances. Most lesions present as firm, well‐circumscribed papules or nodules, typically on the extremities, but their morphology can vary considerably among different histopathological variants. Histologically, DF is characterized by a dermal proliferation of bland spindle or fibrocytic cells, often accompanied by epidermal hyperplasia and basal layer hyperpigmentation. Although its pathogenesis remains incompletely understood, proposed mechanisms include reactive responses to local trauma or inflammation as well as spontaneous development [1–4].

Several histopathological variants of DF have been described, including cellular, aneurysmal, hemosiderotic, epithelioid, sclerotic, and atrophic forms [2, 3]. Recognition of these variants is clinically relevant because their morphology may differ considerably from that of conventional DF and may create diagnostic overlap with other cutaneous neoplasms [3]. Although clinical and dermoscopic findings may provide useful clues, histopathological examination remains important when the clinical appearance is atypical or concerning [3, 5].

Atrophic DF is an uncommon variant characterized clinically by a depressed or indented appearance and histologically by attenuation of the dermal component [5–7]. Unlike the typical firm papule or nodule of conventional DF, the atrophic variant may present as a depressed patch or plaque and can resemble other atrophic, scar‐like, or neoplastic lesions [5–7]. Recent reports have also demonstrated variability in its clinical and dermoscopic presentation, emphasizing the importance of histopathological correlation in atypical lesions [5].

In this report, we describe a 35‐year‐old woman with a long‐standing hyperpigmented, depressed plaque on the back that was diagnosed as atrophic DF. We describe the clinical, histopathological, and immunohistochemical findings and place the case in the context of previously reported examples of this uncommon variant.

## 2. Case Presentation

A 35‐year‐old Saudi female presented with a solitary, hyperpigmented, depressed plaque on her back, which she had noticed for the past five years. The lesion initially began as a small papule and gradually increased in size over time. Despite its progression, the patient did not report any associated symptoms, such as pain or itching, or any systemic complaints. She had no relevant history of previous skin disease, local trauma, insect bites, or other predisposing factors and was not taking any medications.

On physical examination, the lesion measured 1.0 × 0.5 cm and appeared as a well‐demarcated, hyperpigmented, depressed plaque on the back, without ulceration, discharge, or surrounding erythema. A positive dimple sign was noted on examination (Figure 1).

**FIGURE 1:**
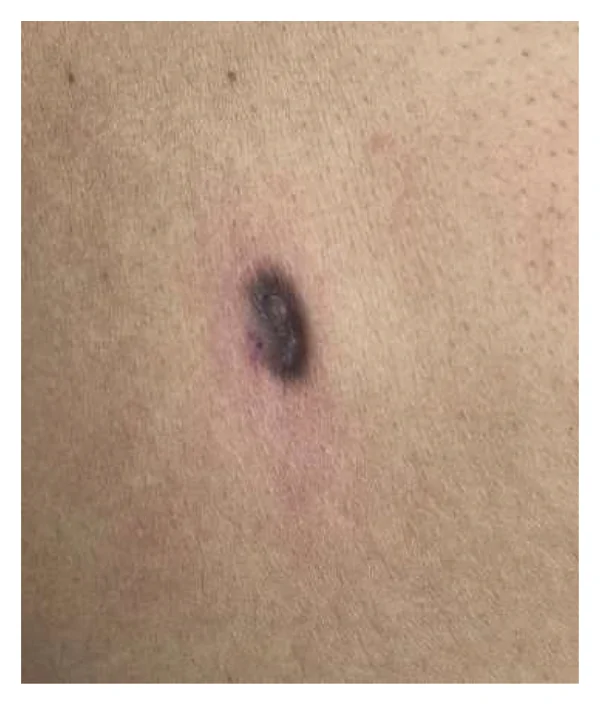
Clinical image showing a well‐demarcated, hyperpigmented, and depressed plaque located on the back.

A punch biopsy was performed to further evaluate the lesion. Histopathological examination demonstrated an uncapsulated, relatively well‐demarcated dermal lesion composed of bland spindle cells arranged predominantly in a storiform pattern and interspersed among collagenous fibrous bundles. The spindle cells exhibited elongated, tapered nuclei with pointed ends and scant eosinophilic to pale cytoplasm. No significant nuclear atypia or pleomorphism was identified, and mitotic figures were infrequent. Touton‐type giant cells were present, with no necrosis identified. The overlying epidermis showed epidermal hyperplasia with basal layer hyperpigmentation. A Grenz zone was present, separating the epidermis from the underlying dermal proliferation. Focal collagen entrapment was identified at the periphery of the lesion. The proliferation involved the dermis without significant extension into the subcutaneous adipose tissue (Figure 2).

**FIGURE 2:**
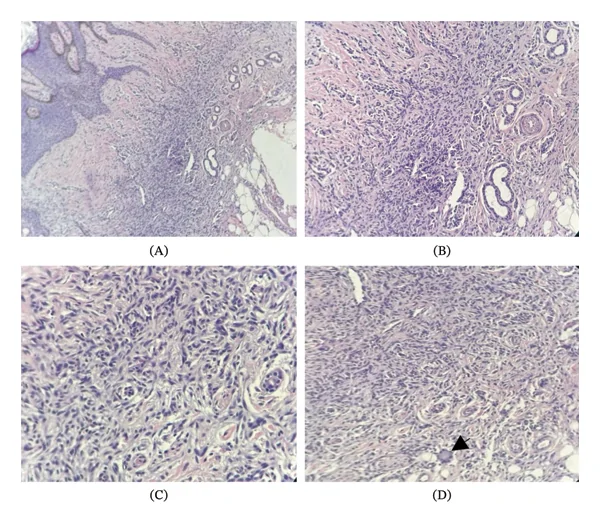
Histopathological examination with hematoxylin and eosin (H&E) staining. (A, B, and C) Dermal lesion composed of bland spindle cells interspersed among fibrous bundles (H&E; 4×, 10×, and 20×). (D) Touton‐type giant cells (arrow) (H&E; 40×).

The lesion demonstrated relative thinning of the dermis with preservation of the characteristic storiform architecture and bland cytologic features, supporting the diagnosis of an atrophic variant of DF. The absence of significant cytologic atypia, pleomorphism, increased mitotic activity, necrosis, or a destructive infiltrative growth pattern further supported a benign fibrohistiocytic lesion.

Immunohistochemical studies were performed using CD34, Factor XIIIa, S100, HMB45, and p63. The spindle cells showed focal immunoreactivity for Factor XIIIa and were negative for CD34, S100, HMB45, and p63. The focal Factor XIIIa expression, in conjunction with the characteristic histomorphologic features, supported the diagnosis of DF. Verhoeff–van Gieson staining was not performed because it was not available; therefore, loss of elastic fibers could not be assessed in the present case. Overall, the combination of the characteristic histomorphology and supportive immunophenotype was consistent with an atrophic variant of DF.

Following the biopsy and establishment of the diagnosis, the patient was advised regarding the benign nature of the lesion and appropriate clinical follow‐up. However, the patient was subsequently lost to follow‐up, and no further clinical outcome or recurrence assessment was available.

## 3. Discussion

DF is a common benign fibrohistiocytic neoplasm that typically presents as a firm papule or nodule. Histologically, conventional DF shows a dermal proliferation of bland spindle or fibrocytic cells, often arranged in a storiform pattern, with variable collagen entrapment and epidermal changes. Factor XIIIa expression and lack of CD34 expression may support the diagnosis, although immunohistochemical findings are variable and should be interpreted with the histomorphology [3, 8, 9]. Atrophic DF is an uncommon variant characterized by a depressed clinical appearance and relative attenuation of the dermal component [6, 7]. Unlike conventional DF, in which the dermal proliferation usually produces a palpable papule or nodule, the reduced dermal component in atrophic DF may result in a subtle depressed plaque or macule.

Reported cases demonstrate considerable clinical and histopathological variability (Table 1). Atrophic DFs have been described as depressed plaques, macules, nodules, and linear depressions at different anatomical sites [6, 10–16]. Histopathological findings reported across these cases include dermal thinning, spindle‐cell proliferation, storiform architecture, epidermal hyperplasia with basal hyperpigmentation, collagen entrapment, and variable loss of elastic fibers [6, 11–16]. Dermoscopic findings are also variable, including pigment networks, scar‐like white areas, and vascular structures [13, 16]. The present case shares several of these reported features and further illustrates the variable clinical and histopathological spectrum of this uncommon variant.

**TABLE 1:** Summary of reported cases of atrophic dermatofibroma and their clinicopathologic features.

Author and year	Age/sex	Clinical presentation	Site	Histopathological features	IHC/ancillary findings
Kusano et al., 2024 [10]	47/M	Asymptomatic 5‐mm brownish dermal nodule with central depression, present for 5 years	Left thigh	Central depression with dermal atrophy; proliferation of fibroblastic tumor cells in the dermis and hyperplasia of the overlying epidermis	Factor XIIIa positive; CD34 negative; decreased elastic fibers on Elastica van Gieson staining; intense MMP‐2 expression

Puri et al., 2021 [11]	29/F	Asymptomatic blackish‐to‐brownish indurated plaque, 0.5–1 cm, present for 7 years; positive dimple sign	Left lower leg	Nonencapsulated dermal tumor extending from the upper to deep reticular dermis and superficial subcutis; epidermal hyperplasia with slight central epidermal atrophy; intertwined/interwoven spindle cells; collagen trapping	Factor XIIIa positive; CD34 negative; reduced elastic fibers on van Gieson staining

Gutierrez et al., 2021 [12]	47/M	Flat, atrophic, hyperpigmented 5 × 5 mm macule	Right upper back	Epidermal acanthosis, basilar hyperpigmentation, fibroblast proliferation, and trapped collagen bundles; loss of elastic fibers	VVG staining demonstrated loss of elastic fibers; no IHC findings reported for this case
	68/M	Depressed, erythematous 6 × 5 mm patch	Left posterolateral shoulder	Epidermal acanthosis, basilar hyperpigmentation, dermal fibroblast proliferation/focal fibroplasia, and trapped collagen bundles; absence of elastic fibers within the lesion	VVG staining demonstrated absence of elastic fibers; no IHC findings reported for this case

Aktaş Karabay et al., 2021 [13]	54/F	Depressed reddish lesion; dermoscopy showed a peripheral pigment network, pink‐red coloration, central whitish scar‐like patch, and white‐yellow scales	Back	Histopathological findings described as typical of atrophic dermatofibroma	Dermoscopy demonstrated the findings described above; exact IHC findings were not available in the accessible report

Yano‐Takamori et al., 2019 [14]	45/M	Brown spot approximately 1 cm in diameter with an atrophic, recessed surface	Right shoulder	Remarkable dermal atrophy with a well‐defined spindle‐cell proliferation; small nuclei without atypia; subcutaneous adipose tissue immediately beneath the atrophic dermis	Factor XIIIa positive; CD34 negative; elastic fibers disappeared or were greatly decreased on Elastica van Gieson staining; strong MMP‐1 expression in the atrophic dermis

Takafuji et al., 2019 [15]	25/F	Linear concavity noticed 1 year previously	Right lateral back	Fibrous and histiocytic tumor in the deep dermis; spindle‐to‐rhomboid tumor cells arranged in an irregular storiform pattern; Touton giant cells and histiocytic cells centrally; tumor extended toward fatty tissue	Factor XIIIa positive; CD34 and CD68 negative; almost complete loss of elastic fibers on Elastica van Gieson staining

Villarreal‐Martinez et al., 2016 [16]	40/F	Nontender 1.5 cm well‐defined erythematous atrophic plaque; dermoscopy showed pigment network, scar‐like white patches, and arborizing vessels	Left upper arm	Marked thinning of the dermis with epithelioid and spindle cells in a storiform pattern; aneurysmal features	Factor XIIIa focally positive; CD34 positive in vascular endothelium; arborizing vessels on dermoscopy

Present case	35/F	Solitary asymptomatic hyperpigmented depressed plaque, present for 5 years; positive dimple sign	Back	Relatively well‐demarcated dermal spindle‐cell proliferation with storiform architecture; epidermal hyperplasia and basal hyperpigmentation; collagen entrapment; Touton‐type giant cells; no significant subcutaneous extension	Factor XIIIa focally positive; CD34, S100, HMB45, and p63 negative; VVG staining not performed

*Note:* DF, dermatofibroma; IHC, immunohistochemistry; MMP‐1, matrix metalloproteinase‐1.

Abbreviations: CD34, cluster of differentiation 34; MMP‐2, matrix metalloproteinase‐2; VVG, Verhoeff–van Gieson.

The main histopathological differential diagnosis is atrophic dermatofibrosarcoma protuberans (DFSP), which may have a similar depressed clinical appearance. Atrophic DF generally retains a relatively circumscribed architecture, whereas atrophic DFSP shows a deeper and more irregular infiltrative pattern, particularly with extension into subcutaneous adipose tissue [6, 17]. Zelger et al. found that most of 26 atrophic DFs were CD34‐negative with variable Factor XIIIa expression, whereas all three atrophic DFSPs were CD34‐positive and Factor XIIIa–negative [17]. Other mimics, including melanocytic lesions, basal cell carcinoma, and scar tissue, can be distinguished by their respective cellular lineage and architectural features. Thus, the diagnosis of atrophic DF requires correlation of the residual features of DF with the overall architecture and appropriate ancillary studies rather than relying on dermal thinning alone [3, 6, 17].

Factor XIIIa positivity in the present case was focal and therefore supportive rather than diagnostic. This is consistent with the variable expression reported in atrophic DF. In the series of Zelger et al., Factor XIIIa was expressed in 20 of 26 atrophic DFs, with greater expression observed in lesions considered to represent an earlier stage [17]. Accordingly, focal Factor XIIIa expression should be interpreted together with the characteristic histomorphology and CD34 negativity rather than as an isolated diagnostic marker.

The pathogenesis of atrophic DF remains uncertain. Loss or reduction of elastic fibers has been reported in several cases and proposed as a possible contributor to the depressed morphology [6, 11–16]. However, this finding is not consistently demonstrated. In addition, Yano‐Takamori et al. reported increased matrix metalloproteinase‐1 expression, while Kusano and Yamamoto reported increased matrix metalloproteinase‐2 expression in separate cases [10, 14]. These observations raise the possibility that extracellular matrix remodeling may contribute to dermal attenuation, but the evidence is limited to individual reports and does not establish a common mechanism. Verhoeff–van Gieson staining was not available in the present case, so elastic‐fiber alteration could not be assessed. Overall, recognition of atrophic DF requires integration of its clinical appearance with the characteristic, although sometimes attenuated, histopathological features and appropriate ancillary findings.

## Author Contributions

Renad F. Althobaiti: conceptualization, investigation, data curation, writing–original draft, supervision, and writing–review and editing.

Jana Khalid Alharbi: conceptualization, investigation, data curation, and writing–original draft.

Asmaa Alabbadi: conceptualization, investigation, data curation, and writing–original draft.

Dai Zafer: conceptualization, supervision, and writing–review and editing.

Razan Alluhaibi: conceptualization, investigation, data curation, and writing–original draft.

Bayan Zakaria Hafiz: conceptualization, supervision, and writing–review and editing.

## Funding

This study did not receive any financial support.

## Disclosure

All authors have read and approved the final version of the manuscript.

## Ethics Statement

Ethical approval was not required for this study in accordance with local and national guidelines. This case report was reviewed, and the need for approval was waived by the Institutional Review Board of the Ministry of Health, Kingdom of Saudi Arabia.

Written informed consent was obtained from the patient for publication of the details of their medical case and any accompanying images.

## Consent

Written informed consent was obtained from the patient for publication of this case report and accompanying images.

## Conflicts of Interest

The authors declare no conflicts of interest.

## Supporting Information

Additional supporting information can be found online in the Supporting Information section.

## Supporting information


**Supporting Information** Compliance with reporting guidelines. This manuscript has been prepared in accordance with the CARE Checklist for case reports (see Form Section).

## Data Availability

The data that support the findings of this study are not publicly available due to patient privacy but are available from the corresponding author, Renad Althobaiti, upon reasonable request.
