# Supplementary material for: Atrophic Dermatofibroma: A Rare Variant With Distinct Clinical and Histopathological Features
Source: Case Rep Dermatol Med. 2026 Oct 21;2026:4419249. doi: 10.1155/crdm/4419249 (PMC13685498; doi:10.1155/crdm/4419249)
Supplement: Supplementary file 1 [file CRDM-2026-4419249-s001.pdf]

CARE Checklist of information to include when writing a case report

Topic	Item	Checklist item description	Reported on Line
**Title**	**1**	The diagnosis or intervention of primary focus followed by the words "case report"	Line 1
**Key Words**	**2**	Two to five key words that identify diagnoses or interventions in this case report, including "case report"	Lines 62–63
**Abstract (no references)**	**3a**	Introduction: What is unique about this case and what does it add to the scientific literature?	Lines 39–45
	**3b**	Main symptoms and/or important clinical findings	Lines 46–55
	**3c**	The main diagnoses, therapeutic interventions, and outcomes	Lines 46–55
	**3d**	Conclusion—What is the main "take-away" lesson(s) from this case?	Lines 56–60
**Introduction**	**4**	One or two paragraphs summarizing why this case is unique (may include references)	Lines 73–88
**Patient Information**	**5a**	De-identified patient specific information	Lines 92–93
	**5b**	Primary concerns and symptoms of the patient	Lines 92–95
	**5c**	Medical, family, and psycho-social history including relevant genetic information	Not reported
	**5d**	Relevant past interventions with outcomes	Not reported
**Clinical Findings**	**6**	Describe significant physical examination (PE) and important clinical findings	Lines 96–98
**Timeline**	**7**	Historical and current information from this episode of care organized as a timeline	Lines 92–94
**Diagnostic Assessment**	**8a**	Diagnostic testing (such as PE, laboratory testing, imaging, surveys)	Lines 99–107
	**8b**	Diagnostic challenges (such as access to testing, financial, or cultural)	Lines 109–112
	**8c**	Diagnosis (including other diagnoses considered)	Lines 105–107, 113–116
	**8d**	Prognosis (such as staging in oncology) where applicable	Lines 83–85
**Therapeutic Intervention**	**9a**	Types of therapeutic intervention (such as pharmacologic, surgical, preventive, self-care)	Lines 99, 143–144
	**9b**	Administration of therapeutic intervention (such as dosage, strength, duration)	Not reported
	**9c**	Changes in therapeutic intervention (with rationale)	Not applicable
**Follow-up and Outcomes**	**10a**	Clinician and patient-assessed outcomes (if available)	Not reported
	**10b**	Important follow-up diagnostic and other test results	Not reported
	**10c**	Intervention adherence and tolerability (How was this assessed?)	Not applicable
	**10d**	Adverse and unanticipated events	Not reported
**Discussion**	**11a**	A scientific discussion of the strengths AND limitations associated with this case report	Lines 109–151
	**11b**	Discussion of the relevant medical literature with references	Lines 109–151
	**11c**	The scientific rationale for any conclusions (including assessment of possible causes)	Lines 113–116, 143–151

Topic	Item	Checklist item description	Reported on Line
	**11d**	The primary "take-away" lessons of this case report (without references) in a one paragraph conclusion	Lines 154–161
**Patient Perspective**	**12**	The patient should share their perspective in one to two paragraphs on the treatment(s) they received	Not reported
**Informed Consent**	**13**	Did the patient give informed consent? Please provide if requested	Lines 261–262, 268–269 — Yes

CARE 2013 — https://www.care-statement.org | CC BY-NC-ND
